# Student Perceptions of Academic Engagement and Student-Teacher Relationships in Problem-Based Learning

**DOI:** 10.3389/fpsyg.2021.713057

**Published:** 2021-10-28

**Authors:** Carmen M. Amerstorfer, Clara Freiin von Münster-Kistner

**Affiliations:** Department of English, University of Klagenfurt, Klagenfurt, Austria

**Keywords:** academic engagement, communication style, feedback, problem-based learning, student-teacher relationships, teacher caring, teacher credibility

## Abstract

Students’ academic engagement depends on a variety of factors that are related to personal learner characteristics, the teacher, the teaching methodology, peers, and other features in the learning environment. Components that influence academic engagement can be cognitive, metacognitive, affective, social, task-related, communicative, and foreign language-related. Rather than existing in isolated spheres, the factors contributing to an individual’s academic engagement intertwine and overlap. The relationships students cultivate with others are prominent in several of these areas. Positive interpersonal relationships enhance individuals’ enthusiasm for learning (Mercer and Dörnyei, 2020), which benefits sustainable learning success and self-confidence. The relationships between students and teachers and the perceptions students have of their teachers seem to be particularly influential on students’ engagement in academic undertakings. Problem-based learning (PBL), a teaching approach particularly suitable for tertiary education, involves students in authentic problem-solving processes and fosters students’ self-regulation and teamwork. Intensive relationship-building is one of the key characteristics of this student-centered approach (Amerstorfer, 2020). The study reported in this article explores the connection between the academic engagement of 34 students and their perceptions of three instructors in a teacher education program for pre-service English teachers in Austria. An online questionnaire was used to investigate the participants’ perceived academic engagement (effort, dedication, learning success) in a university course that implements PBL as its underlying teaching methodology in comparison to conventional teaching approaches. The study further examines how the students perceived the course instructors’ caring, credibility, communication style, and feedback, which leads to new information about how PBL shapes student-teacher relationships. Due to Covid-19, the otherwise face-to-face course was taught online.

## Introduction

Academic engagement happens when students dive deep into learning activities, when they are mentally and emotionally absorbed by the study materials, and often when interacting with peers. Academic engagement goes beyond “surface learning” (Hattie, 2003, p. 9) like content memorization and fulfilling requirements to achieve a passing grade for a course. It draws students into intense thinking activities like analyzing and understanding concepts, rationalizing procedures, and deducing meaning. It involves social interaction with peers and the teacher, in the form of exchanging experiences, knowledge, opinions, and support. Problem-based learning (PBL) requires both academic engagement and intense peer interaction. It changes the relationships between students and teachers in comparison to conventional teaching approaches in tertiary education. This article explores how PBL shapes the academic engagement of preservice English teachers and their relationships with the course instructors.

## Literature Review

### Academic Engagement in Tertiary Education

Much of the literature about learner engagement refers to students in primary and secondary education. While featuring terminological idiosyncrasies, several models of learner engagement recognize similar physiological, behavioral, and psychological components (Marks, 2000; Finn and Zimmer, 2013; Skinner and Pitzer, 2013). Linnenbrink and Pintrich (2003), for instance, discuss the academic engagement of students in terms of behavioral engagement (i.e., effort, persistence, instrumental help-seeking), cognitive engagement (i.e., strategy use, metacognition), and motivational engagement (i.e., interest, value, affect). Academic engagement at tertiary level has much in common with engagement at primary and secondary levels but it must be defined as a concept of its own due to the overall different context.

From our experience, university students have generally more autonomy in comparison to primary or secondary school pupils; for example, they can choose which courses to attend and have more options regarding participation. In return, they are more expected to manage their own study progress and regulate their own learning. The gained autonomy, thus, causes increased responsibility. Furthermore, it seems that the expectations in young adults of society and individuals in a student’s immediate environment (e.g., family members) are more sophisticated and often linked to cultural norms. Individual students can experience the transition from earlier to tertiary education as cognitively and emotionally challenging. The changes can cause negative repercussions on the self-concept and related psychological features of individuals, such as resilience and anxiety. For instance, compared to university students, young children do not consider it a weakness to ask the teacher for help (Brooks et al., 2013). From our experience, university students, on the other hand, often hesitate and consider what their peers will think of them if they reveal that they do not understand or know something. Also, the social standing in a group gains importance, and particularly adolescents and young adults can be troubled with internal battles regarding their self-confidence. What is more, university students are more mature in regard to their cognitive, social, emotional, and behavioral development. They have developed certain expectations in their own education, and their personal interests have evolved since childhood. University students, in comparison to school pupils, usually have more clearly defined aspirations and matured personality characteristics. In sum, the transition from childhood to adulthood is accompanied by numerous changes and challenges that can affect students’ academic engagement.

In light of the altered contextual and individual conditions of students in tertiary education and on the basis of literature reviews and personal teaching experience, we globally define academic engagement as all student behavior related to planning, managing, and completing their university education. We identify the following components as essential for academic engagement:

• ***Cognitive engagement*** comprises all kinds of thinking activities related to the involvement and participation in academic tasks, for example, paying attention; acquiring, processing, and storing information; as well as retrieving information from memory.
• ***Metacognitive engagement*** describes the behavior students apply to manage and reflect on their cognitive actions. It includes short-term and long-term planning; coordinating learning tasks; evaluating learning progress and outcomes; and compensating for knowledge gaps.
• ***Affective engagement*** is what students do to regulate their own and their peers’ emotions. It includes handling boredom and curiosity; acknowledging and controlling anxieties; evaluating, generating, and maintaining interest and motivation; as well as demonstrating empathy toward others.
• ***Social engagement*** comprises different forms of interaction with fellow students and teachers. It includes establishing a facilitative network of peers and teachers; cultivating supportive relationships with individuals; contributing to group efforts; and being available for others in need.
• ***Task engagement*** is the manner and intensity with which students engage with learning materials in meaningful ways. It is strongly influenced by an individual’s interest and motivation and also depends on other personal attributes, such as resilience and endurance. Task engagement includes practicing academic skills as well as setting obtainable goals and prospective rewards.
• ***Communicative engagement*** is what students do to effectively communicate with others in writing, speaking, and non-verbally. It includes receptive activities (e.g., attentive listening; observing body language, gestures, and facial expressions) and productive activities (e.g., building and presenting arguments; refuting the arguments of others; agreeing and disagreeing). Patience and respect play important roles in communicative engagement.

The university course described in this article is conducted in English, which is a foreign language for the students. Therefore, language engagement (Svalberg, 2018) must also be considered part of academic engagement in the current study. Language engagement is strongly connected to communicative and affective engagement and adds to the complexity of academic engagement through considerations regarding the students’ self-concept (Mercer and Williams, 2014) and individuality (Gregersen and MacIntyre, 2014) as users of English as a foreign language (EFL). Hence, we include an additional component in our model of academic engagement.

• ***Foreign language engagement*** is characterized by students’ efforts involved in using a foreign language for academic purposes. It comprises general language skills (i.e., being able to read, listen, write, and speak in the foreign language); linguistic knowledge and ability (e.g., vocabulary, spelling, pronunciation); metalinguistic awareness (e.g., academic style; tone of voice; contextual appropriateness; cultural and regional variation); and psychological aspects (e.g., foreign language anxiety, willingness to communicate).

The individual components of academic engagement must not be regarded as isolated features as there is much overlap (e.g., between metacognitive and task engagement when planning how to approach and complete a task). The components are tightly intertwined and influenced by students’ knowledge (e.g., subject-specific knowledge; knowledge of cultural norms), skills (e.g., strategic planning; composing academic text), and abilities (e.g., being able to empathize with others; linguistic abilities). Unlike other models of engagement that have inspired our model (e.g., Finn and Zimmer, 2013^1^), we prefer to think of academic engagement in tertiary education as the *overarching* issue that depends on the components that constitute it.

In general, teachers want students to engage deeply in study activities because students’ dedication and effort have a positive effect on learning success and achievement (Linnenbrink and Pintrich, 2003; Christenson et al., 2013; Mercer and Dörnyei, 2020). Teachers are in a position in which they can shape the engagement of students (Skinner and Pitzer, 2013) by creating a facilitative, motivating learning environment. Mercer and Dörnyei (2020), for instance, recommend the “Socratic method” for teaching, that is asking questions to promote critical thinking, as well as getting students to prepare questions for each other, which leads to sustainable and transferrable learning outcomes. Another way to increase academic engagement is a discovery approach to generate curiosity by letting students find out answers to questions and solutions to problems by themselves “simply for the reward of the pleasure of knowing more” (Mercer and Dörnyei, 2020, p. 108). Such activities involve students in profound, meaningful thinking processes that create knowledge (e.g., by analyzing, comparing, reflecting, and contrasting information) instead of merely consuming knowledge (e.g., by hearing it from the teacher or reading it in a book).

An attractive task design is also beneficial for academic engagement. A task is emotionally captivating if its design is physically appealing and if the students appreciate the type of the activity and its content (Mercer and Dörnyei, 2020). The latter should be meaningful, valuable, and interesting for the students, triggering positive emotions during the learning activity (Linnenbrink and Pintrich, 2003). Moreover, tasks should have a clear focus, enabling students to understand exactly what they are expected to do (Mercer and Dörnyei, 2020). Providing explicit instructions – a teaching act that requires careful planning and smooth delivery in both speaking and writing – is therefore critical.

### Student-Teacher Relationships in Tertiary Education

Student-teacher relationships at university have been less investigated in comparison to elementary, primary, and secondary education (Hagenauer and Volet, 2014). Nevertheless, university students and teachers also cultivate unique relationships with each other, which are positive when characterized by honesty, respect, trust, safety, caring, and support (Fitzmaurice, 2008; Komarraju et al., 2010). Studies that investigate how university students and teachers perceive their relationships (e.g., Anderson and Carta-Falsa, 2002; Fitzmaurice, 2008; Komarraju et al., 2010) often focus on qualitative information, which is also the case in the current study. According to Hagenauer and Volet (2014), student-teacher relationships in higher education are complex and context-dependent. They depend on the frequency and quality of interactions as well as on an affective and a support dimension (ibid.), which refer to the following:

• The *affective* dimension, which describes the bond built between students and teachers, forming the basis for secure and affective positively experienced relationships.
• The *support* dimension, which describes the support that must be provided through TSR [teacher-student relationships] for students’ success at university (e.g., teacher setting clear expectations, answering emails promptly). (Hagenauer and Volet, 2014, p. 374; emphasis added)

The study at hand acknowledges the complexity and multi-dimensionality of the interwoven topics and focuses on the characteristics and actions of teachers within the context of PBL. Specifically, teacher credibility, caring, feedback, and communication style are scrutinized in order to investigate how they might enhance academic engagement and contribute to positive student-teacher relationships.

#### Teacher Credibility and Caring

A core prerequisite for learning is a caring pedagogy with credible teachers who afford a supportive, student-centered classroom environment (Noddings, 1992; Noblit, 1993; Wentzel, 1997; Elizabeth et al., 2008; Pishghadam and Karami, 2017; Duffy, 2018; Pishghadam et al., 2018; Mercer and Dörnyei, 2020). Students who perceive their teachers as credible and caring are more academically engaged, including a higher willingness to take risks and a higher level of persistence when faced with failure (Davis et al., 2012).

Research into teacher credibility began in the 1970s when the classical concept of ethos or source credibility (i.e., “the degree to which a source is perceived to be believable”; Banfield et al., 2006, p. 65) was connected to investigations of instructional communication. Teacher credibility thus refers to the degree to which students find a teacher believable. McCroskey et al. (1974) noticed that teacher credibility depends on five criteria: competence, character, sociability, composure, and extraversion. After further inquiry, McCroskey and Young (1981) proposed that teacher credibility is exclusively limited to two dimensions, a teacher’s competence and character. Additional studies revealed that caring is another fundamental component of teacher credibility. With McCroskey and Teven’s (1999) widely used, three-dimensional assessment instrument for teacher credibility, students evaluate their perception of a teacher’s credibility according to the teacher’s competence (intelligence, training, expertise, knowledgeability, competence, brightness), goodwill (care about student, care about student’s interest, self-centeredness, concern with student, sensitivity, understanding), and trustworthiness (honesty, honorability, morale, ethics, genuineness).

Reviewing related academic literature, Finn et al. (2009) noticed that research has so far focused on three topic areas related to teacher credibility: its effect on student outcomes and learning; its effect on instructional communication processes; and teacher characteristics and communication behaviors that foster credibility. Teachers who are perceived as credible by their students use argumentative messages; demonstrate verbal and non-verbal immediacy behaviors; seek affinity with students; appropriately use technology for teaching; are assertive and responsive; and engage with their students outside of class (consult Finn et al., 2009 for a detailed literature review with references). Teacher credibility has further been found to promote effective student-teacher communication and relationships. Also increased student motivation and positive learning outcomes have been noted (Finn et al., 2009).

Together with teacher credibility, caring is believed to be one of the key characteristics of effective teachers (Elizabeth et al., 2008; Pishghadam and Karami, 2017; Pishghadam et al., 2018). Similar to academic engagement (see section “Academic engagement in tertiary education”), most research about teacher caring has been conducted in primary and secondary education. Nevertheless, there are some texts about caring in adult education (e.g., Watson, 2008, 2018; Duffy, 2018; Motta and Bennett, 2018), which support our hypothesis that despite contextual differences and students’ progressed cognitive, social, and emotional development, teacher caring is important for academic engagement and relationship-building at tertiary level, too. The implementation of a caring pedagogy is influenced by factors like class size, teaching format (i.e., face-to-face or remote teaching), and teachers’ ethical beliefs about pedagogy and caring (Watson, 2008, 2018; Christopher et al., 2020). Below we scrutinize the traits and actions of caring teachers situated in a university context, many of which can also be attributed to teacher credibility, its inseparable spouse.

##### Caring Teachers Pay Attention to Teaching and Learning

Caring teachers invest time and effort into the preparation of their teaching and manage it in a student-centered, democratic fashion. They adapt the syllabus within the given study program to cater for the students’ preferences and thoughtfully integrate reading materials and other sources according to the students’ interests. Caring teachers conduct communicative learning activities that engage students in collaborative problem-solving and discussion, which leads to sustained learning outcomes (Strobel and van Barneveld, 2009; Yew and Goh, 2016). Where possible, caring teachers give students choices and autonomy regarding learning contents and procedures, putting students in “positions of genuine responsibility” (Mercer and Dörnyei, 2020, p. 59).

##### Caring Teachers Are Available

Caring teachers are there for students – even beyond the classroom (Ekmekci, 2013). They are available outside class time to answer students’ questions, listen to concerns, or simply engage in casual chats. They inform students about contact options, offer regular or irregular consultation hours, and promptly respond to emails. Social presence is vital for signaling care (Plante and Asselin, 2014), which may be more complicated though not less important in remote learning than face-to-face. In online instruction, caring teachers can use online learning platforms, social media apps, email, and websites for out-of-class communication. Switched-on cameras during online class meetings contribute to a sense of physical and social closeness and foster “student wellbeing through virtual presence” (Christopher et al., 2020, p. 823). Whether online or in person, by making themselves available, caring teachers demonstrate genuine interest in their students through simply being there.

##### Caring Teachers Provide a Psychologically Safe Learning Environment

Caring teachers convey feelings of closeness, understanding, and appreciation to students. They have high respect for students, demonstrate commitment for them, and are receptive to student needs (Hattie, 2003). They create a climate of mutual care and trust (Noddings, 1992) in order to facilitate open, democratic communication (Ranson, 2018). They seek mutual understanding and perspective taking with students (Noddings, 1992) and foster interaction with and among students that is characterized by the same values. Caring teachers promote positive group dynamics (Dörnyei and Murphey, 2003; Williams et al., 2015) and enforce classroom conduct that is free of ridicule, blame, and embarrassment. They nurture an encouraging and supportive learning environment (Hawk and Lyons, 2008), in which students feel valued and safe.

##### Caring Teachers View Students as Individuals With Personal Characteristics

Caring teachers try to establish a holistic perspective of individual students (Motta and Bennett, 2018). They appreciate students’ uniqueness as individuals, as members of society, and as learners in class (Hult, 1980; Hawk and Lyons, 2008). Caring teachers recognize students’ personal strengths and weaknesses and “use their professional and moral judgment in responding” appropriately (Noddings, 2012, p. 774). Discovering students’ uniqueness involves paying attention to and analyzing psychological characteristics such as self-concept, beliefs, affect, motivation, and agency (Williams et al., 2015). Caring teachers appraise students’ learning style and strategy preferences and understand how individuals function in small teams and in the class community. Furthermore, they account for students’ personal characteristics when planning the syllabus and teaching materials.

##### Caring Teachers Are Empathetic

Caring teachers are “emotionally intelligent” (Goleman, 1996) and eager to understand their students’ perspectives. They cultivate meaningful dialogs with students to establish relations of care and trust, which helps to “achieve empathic accuracy” (Noddings, 2012, p. 775). Caring teachers are attentive to students’ verbal remarks regarding their emotional state and try to decode non-verbal cues such as facial expressions, body language, and eye contact (Mercer, 2016; see also for theory of empathy). They listen and observe without judgment (Mercer, 2016), try to understand students’ thoughts and feelings (Mercer and Dörnyei, 2020), and display “compassion and tender-heartedness” toward their students (Oxford, 2016, p. 18).

##### Caring Teachers Foster Development Beyond Content-Related Aspects

Caring teachers are committed to both the academic and personal development of their students (Hattie, 2003). They expect and encourage students to do their best within their abilities (Noddings, 1992). They convince students that temporary failure is inevitable, included in most learning, and that making mistakes is an important part of the process. Caring teachers help students develop strategies to handle setbacks and frustrations to avoid the manifestation of any associated negative emotions in a student’s self-concept. They help students set effective goals, boost their self-confidence and motivation, and aim for the development of growth mindsets (Hattie, 2003; Ryan and Mercer, 2012; see section “Teacher feedback and communication style”).

##### Caring Teachers Build Interpersonal Relationships With Students

Caring teachers cultivate genuine relationships with their students. They know their students’ names and can pronounce them correctly, which signals respect and creates a feeling of belonging (Bonwell and Eison, 1991; Mercer and Dörnyei, 2020). Communicative classrooms provide plenty of opportunities for teachers to learn about students’ opinions, attitudes, beliefs, and interests. Caring teachers signal curiosity in their students’ lives beyond the classroom and offer occasional insights into their own lives as well. Getting to know others, appreciating their stories, and enjoying their company can be pleasurable for both sides. It can lead to strong interpersonal relationships between students and teachers, which positively affect learner engagement (Rubie-Davies, 2015; Mercer and Dörnyei, 2020).

##### Caring Teachers Foster Positive Peer Relationships

Additional to their own relationships with students, caring teachers foster supportive peer relations in order to encourage academic engagement. The aim is that “everyone feels accepted, valued, safe and included in group life” (Mercer and Dörnyei, 2020, p. 71) with a perceived reciprocal importance between individual students and the group (Dewaele and MacIntyre, 2016). Caring teachers use their positions to steer group dynamics and socio-emotional processes in class toward mutual understanding and support among students. The gained sense of safety, trust, and belonging consequently leads to less conflict, more cooperation and engagement, and overall increased student wellbeing (Hart and Hodson, 2004).

##### Caring Teachers Are Good Communicators

Caring teachers are expert listeners and observers. Instead of assuming student needs, they try to recognize and understand the needs actually expressed during student interaction in the classroom (Noddings, 2012). Caring teachers involve students in conversations and ask specific questions about their needs. Furthermore, they clearly state what is expected of the students in terms of learning and performance (Hawk and Lyons, 2008). Caring teachers speak in a pleasant voice, write emails in a respectful tone, convey clear messages, and signal a willingness to elaborate on follow-up issues. Non-verbal communication is also extremely important, though often underrated. A teacher’s genuine smile can be reassuring for students, making them feel at ease, and giving them confidence in their abilities. Caring teachers use their body language to indicate openness, curiosity, and patience. (see section “Teacher feedback and communication style”).

##### Caring Teachers Give Constructive Feedback

Caring teachers recognize the effort students invest during academic engagement. They observe and evaluate both the learning processes and outcomes and consider what kind of feedback would support the learning growth and well-being of individuals. Caring teachers express respect and appreciation in their feedback and always include motivational comments.

The feedback itself should be specific and concrete (Hawk and Lyons, 2008), free of personal judgment, and fairly distributed among students. (see section “Teacher feedback and communication style”).

##### Caring Teachers Provide Feedback Opportunities for Students

In addition to involving students in planning processes, caring teachers give students a voice in retrospect to taught lessons or courses (Hawk and Lyons, 2008). They provide opportunities for students to express their opinions regarding course content, structure (e.g., weekly or blocked lessons), format (e.g., in class, online, hybrid), teaching methodology, and the teacher’s individual teaching and communication style. Collecting feedback can take various forms with pros and cons attached to them. A round of verbal feedback in a group discussion during class might inhibit some students to speak their minds freely. Anonymous written feedback, perhaps online, generally provides more anonymity unless the class size is so small that the teacher can guess who wrote which comments. Caring teachers consider the circumstances and emphasize that all feedback is voluntary and irrelevant to grading. They further choose a suitable time for student feedback. If they aim to make immediate adaptations during a course, they invite student feedback while the course is ongoing. End-of-term feedback can be useful for revising the syllabus and course materials overall. In any case, student feedback is invaluable for caring teachers and providing feedback opportunities for students is a sign of respect, appreciation, and trust toward them.

##### Caring Teachers Model Caring Behavior to Students

Caring teachers are role models who practice an ethic of care through “dialog, listening, modeling, providing practice, and attributing the best motives” to students (Hawk and Lyons, 2008, p. 322; see also Christopher et al., 2020). They display the particular skills, knowledge, and dispositions that characterize them as caring. The aim is to demonstrate the competences and actions of a caring teacher and to support students in developing similar traits.

##### Caring Teachers Look After Their Own Well-Being

Caring teachers regularly reflect on their own well-being and set appropriate actions to create and maintain a high level of long-term well-being. Specifically, they consider how comfortable they feel in their roles as teachers and their workplaces; how healthy they are and what is affecting their physical and psychological health; and how happy they are with life in general and at work (Mercer and Gregersen, 2020). Caring teachers listen to the signals of their body and mind and learn how to react appropriately. They are aware of their individual strengths and weaknesses and focus on continued personal and professional growth (Mercer and Gregersen, 2020).

#### Teacher Feedback and Communication Style

Teacher feedback markedly influences student learning and achievement (Hattie, 2003). It shows students the gap between the current reality and the potential goals or expectations (Riordan, 2021). Frequent, constructive feedback can increase academic engagement (Gettinger and Walter, 2013) if it provides students with useful, comprehensible information about their efforts and directs them toward their learning goals (Mercer and Dörnyei, 2020) in an acceptable and motivating manner.

In general, teacher feedback can focus either on the person (abilities, personality, or character) or on the performance (observable actions or behaviors) (Riordan, 2021). This is a relevant distinction for teachers as the direction of feedback can have severe consequences for individuals. Person-focused feedback can promote a fixed mindset, while behavior-focused feedback can foster a growth mindset (Dweck, 2006). Individuals with a growth mindset believe that they can improve their abilities through effort and practice. Individuals with a fixed mindset believe that they possess the abilities with which they are born and cannot change or improve them. Students are usually somewhere on a continuum between these two extremes (Dweck, 2006; Ryan and Mercer, 2012). As educators, it should be our goal to support our students in developing growth mindsets as learners with a growth mindset are

more likely to be motivated to seek out challenges and to look for opportunities to learn through the adoption of learner-oriented goals. They […] experience more positive emotions and make more adaptive attributions for poor performance that contribute to higher expectations for the future, in turn enhancing motivation. (Ryan and Mercer, 2012, p. 76).

In brief, if feedback focuses on performance, learners with a growth mindset can use it to improve their actions and abilities. On the other hand, feedback focused on personality may lead learners to doubt their overall ability to learn, which may consequently hamper their motivation and foster a fixed mindset – an undesired outcome of teacher feedback.

Performance feedback should relate to the process rather than the outcomes of students’ work because process feedback “is more conducive to behavior change and immediate course correction, whereas outcome feedback feels like a final evaluation” (Riordan, 2021, p. 15). Process feedback can stimulate academic engagement (Shernoff, 2016) and can positively affect students’ self-efficacy by making visible the impact of their efforts (Guthrie et al., 2013; Riordan, 2021). Experiencing success in a learning situation and receiving positive feedback on the processes that led to the success can consequently bolster the self-confidence (i.e., believing in one’s abilities) and self-esteem (i.e., overall self-worth) of individuals. Negative feedback, on the other hand, can have the reverse effect and may cause students to “react defensively […], to reject or actively avoid feedback, and to opt not to use it” (Mercer et al., 2012, p. 18). Mercer and Dörnyei (2020, p. 63) recommend that teacher feedback should focus on “highlighting the effort, strategies and approach taken” even if the outcome of a learning activity was negative.

In order to produce the desired effects, feedback should be relevant and useful to the students. Mercer and Dörnyei name three key areas of which at least one must be addressed by the teacher, “the task itself, the process of working on the task, and/or self-regulation competencies for working further on related tasks” (Mercer and Dörnyei, 2020, p. 63). Effective feedback can be characterized as being “detailed, accurate, and immediate, as well as encouraging and supportive” (Gettinger and Walter, 2013, p. 667), and it should be “clear, positive and specific” (Savin-Baden and Howell Major, 2004, p. 100) instead of vague or general. Teachers should further personalize the feedback by using the pronoun “I” (Savin-Baden and Howell Major, 2004), communicate it as soon as possible after a specific performance, and preferably give it in a private, face-to-face situation (Riordan, 2021).

Feedback can also become a regular, interactive process, in which students and teachers both reflect on an individuals’ learning processes and outcomes. The active involvement in feedback tasks gives students a sense of agency (Mercer and Dörnyei, 2020) and increases their academic engagement (Brooks et al., 2013). In a school context, Mercer and Dörnyei (2020, p. 43) suggest “exit tickets” to make learning progresses visible and to actively engage students in self-reflection. Exit tickets are quick self-feedback tasks that invite learners to briefly evaluate their own learning progress at the end of a lesson by considering a few questions (e.g., what the student learned in the lesson and how confident they feel about their abilities in the moment of reflection).

In general, teacher feedback is accepted more easily when the teacher is considered to be credible and trustworthy, that is “possessing the expertise necessary to judge [students’] behavior accurately” (Ilgen et al., 1979, p. 351; see section “Teacher credibility and caring”). Constructive feedback given with care “can help build trust and enhance the relationship” between students and teachers (Riordan, 2021, p. 15). Destructive or unhelpful feedback, on the contrary, can be harmful for student-teacher relationships. Feedback is more likely to be taken to heart and perceived as fair if the relationship between a student and the teacher is intact (Pat-El et al., 2012).

Effective teacher feedback is an example of mindful communication, which can have tremendous effects on teacher-student relationships and students’ self-perception (Mercer and Dörnyei, 2020). Positive teacher communication belongs in all supportive learning environments (Shernoff, 2016). When teachers use language for different purposes in the classroom, they “simultaneously send a range of hidden implicit messages through speech, choice of vocabulary and interactional discourse patterns about the roles and capabilities” of the students and themselves (Mercer and Ryan, 2013, p. 22). Teachers can use their voice and body language to convey their emotional state. They can vary pitch, volume, facial expressions, gestures, and eye contact and should therefore be attentive to much more than just the content of their speech. They should carefully consider their comments and the possible effects they may have on students (Mercer and Ryan, 2013). Denton (2007, p. 1) puts it straight in claiming that language, verbal and non-verbal, is “one of the most powerful tools available to teachers.”

Teachers are trained to use sophisticated communication skills to foster the academic engagement of students. Ideally, they create a comfortable, safe learning environment, in which engaging is easy and unthreatening for students (Mercer and Dörnyei, 2020). They listen actively and use verbal and non-verbal strategies to stimulate discussion and other group processes and to ensure that the students focus on the subject matter. Rather than simply talking to students, teachers should try to engage in a dialog *with* their students. They can adopt a coaching style in their communication by asking guiding questions to prompt students’ thinking, active listening, and letting the students themselves identify issues (Beere and Broughton, 2013; Mercer and Dörnyei, 2020). In this vein, Mercer and Dörnyei advocate the GROW model (Whitmore, 2017), which can be used to support students “in setting their goals (G); reflecting on what the current reality (R) looks like; exploring the options (O) for achieving the goals and desired future outcomes,” and which helps students to plan what they “will (W) do in concrete terms to keep moving forward toward their goal” (Mercer and Dörnyei, 2020, p. 41).

The relationships between university students and teachers are unique and majorly depend on positive communication. The responses and feedback teachers receive from their students are equally important as teacher feedback directed toward students. The current Covid-19 pandemic has highlighted the significance of non-verbal communication. It has demonstrated how challenging communication can become if the non-verbal component is limited or erased. In the recent past, in-person classroom teaching has been increasingly or fully substituted by online teaching, which has tremendously impacted student-teacher relationships (Amerstorfer, 2021). Teacher credibility may also have suffered because its vital elements may have been lost in virtual space. In online classrooms, particularly where no video images are available, teachers sometimes feel like they are teaching into a void (Amerstorfer, 2021). Simultaneously, students may feel excluded or uncertain about the extent to which they are directly concerned by the teacher’s speech. Such negative experiences on both sides obstruct communication and hamper positive student-teacher relationships.

### Problem-Based Learning in Foreign Language Teacher Education

Problem-based learning (PBL) is a collaborative method of instruction, mainly used in tertiary education, which is suitable in a variety of disciplines such as medicine, health sciences, psychology, law, and business (Schmidt, 2012). Through its strong foundation in reality, PBL can also be applied in teacher education (Amerstorfer, 2020), as is the case in the current study. Rooted in social constructivist theories, PBL is founded on the belief that learning happens through the negotiation of knowledge among active, intentional learners (Hmelo-Silver and Barrows, 2006). Students “engage in dialog through shared experiences, interpretations, reflection, and problem solving” (Christopher et al., 2020, p. 825), while the teacher mainly functions as facilitator. PBL lessons center around close-to-life problem scenarios, which are usually open-ended with multiple possible solutions but may include closed-ended sub-tasks like matching or ranking activities. A problem typically raises questions or asks for elaboration (Moust et al., 2007). It usually leads to discussions, motivates students to formulate appropriate learning goals, and stimulates self-directed learning (Amerstorfer, 2020). One of the pedagogical aims of PBL is for students to simultaneously acquire skills and knowledge that are essential for their future profession.

In the teacher education course central to this study (see section “Data collection”), the students apply a 7-step approach to problem-solving (Moust et al., 2007). In the first step, they clarify the problem’s context and any unclear terms in the problem description. Second, they identify the problem itself or multiple problems within the problem scenario. Third, they brainstorm as many ideas as they can without paying attention to the form and exact relevance of what comes to mind. The fourth step entails a thorough problem analysis in which the students structure their ideas and analyze them in depth. During this process, the students consult and make use of their prior knowledge. It is common that further questions arise at this stage, which students might not be able to answer immediately. In step five, they define learning goals, which they translate into home assignments for individuals, small teams, or the whole group. Step six happens in between two class meetings, where students conduct literature research and consult other out-of-class resources, for example, their internship supervisors or other experienced teachers. During the following class meeting, the group continues pursuing their learning goals. They synthesize and apply the newly collected information, and reflect on the quality of their joint findings, which is the final one of the seven steps (Moust et al., 2007). Successful problem-solving in PBL requires cooperative teamwork. If the group is not satisfied with the outcomes of their research, they return and repeat steps five to seven until they are contented with the solution.

During PBL lessons, students assume different roles to contribute to the problem-solving process. In the EFL teacher education course, there are three roles that students can assume: chairperson, scribe, and regular participant. The *chairperson* functions as facilitator and is the person “who moderates discussions, keeps the team on task and makes sure everyone works and has the opportunity to participate and learn” (Savin-Baden and Howell Major, 2004, p. 86). The *scribe* is the timekeeper of the group, who takes notes during the conversations in class and shares the notes on an online learning platform. The student who functions as the scribe in one lesson becomes the chairperson in the subsequent lesson because they are best prepared for chairing follow-up discussions and tasks (Amerstorfer, 2020). The remaining students are *regular participants*, who contribute constructively to discussions, exchange knowledge and experiences, agree on learning goals, and seek out learning resources in between class meetings. The chairperson and the scribe may also contribute their views and knowledge to the group’s discussions.

Rotating the roles in PBL has numerous advantages. For instance, all students are engaged in the classroom activities; they share the responsibilities involved in the learning processes; they can experiment with varying levels of control over their peers and learning processes, which may temporarily take them out of their comfort zones (imagine, for example, a naturally shy student in the role of chairperson or a naturally dominant student as scribe); they practice leading and participating in discussions; and experience being effective parts of a team. Embodying different roles in PBL “encourages interdependence among team members” (Savin-Baden and Howell Major, 2004, p. 87) and contributes to the development of students’ self-concepts.

PBL teachers are often referred to as tutors or facilitators due to their adapted roles in comparison with more traditional, teacher-centered methodological approaches (Hmelo-Silver, 2004; Savin-Baden and Howell Major, 2004; Moust et al., 2007; Filipenko and Naslund, 2016; Ansarian and Teoh, 2018). In the beginning, PBL teachers may utilize verbal or non-verbal cues and strategies to stimulate discussions and keep the students focused on the subject matter, for instance, by using gestures, asking questions, summarizing and monitoring group progress, returning and deflecting questions, and suggesting alternatives (Savin-Baden and Howell Major, 2004). They provide the necessary guidance, feedback, and support until the students understand the step-by-step approach to problem solving and their new responsibilities.

After a short induction phase, the students gradually take over until they have complete control over the problem-solving process (Hmelo-Silver and Barrows, 2006; Hmelo-Silver and Eberbach, 2012). The teacher’s traditional roles are reduced to those of a coach who scaffolds learning processes (Collins et al., 1989; Hmelo-Silver and Barrows, 2006) by raising awareness to diverse perspectives or inspiring critical thinking, which may be “a major departure from the traditional teacher role” (Mercer and Dörnyei, 2020, p. 40). Teacher communication becomes scarcer and the teacher turns into an attentive observer who only intervenes if necessary or demanded (Amerstorfer, 2020).

### Students’ Engagement in Problem-Based Learning

The authentic problem statements in PBL immediately involve students in communal academic activity. The problems are designed to activate students’ prior knowledge and memory of related experiences right from the start (Hmelo-Silver, 2004; Hmelo-Silver and Eberbach, 2012), thereby sparking intrinsic motivation and creating a sense of purpose for individuals. Engaging problems are interesting, realistic, and relevant for the students and their future career (Amerstorfer, 2020). Their complexity and deliberately ill-structured nature (Hmelo-Silver and Eberbach, 2012; Ansarian and Teoh, 2018) trigger flexible, critical thinking and creative solution-seeking.

In PBL, students are not seen as empty vessels waiting to be filled with knowledge. Instead, they are expected to participate actively in truth-seeking, knowledge-building activities inspired by the close-to-reality problems. Each individual already knows something about the problems to be solved from their own secondary education, previous teaching internships, and university studies. Past events in the students’ lives spark new, sustainable learning as students can better “construct [and memorize] new knowledge when they can relate it to what they already know” (Hmelo-Silver and Eberbach, 2012, p. 3). The different circumstances of individuals, their various, partly overlapping experiences, and a pool of diverse viewpoints, beliefs, and opinions are valuable assets for PBL.

The students “are the agents in the [PBL] classroom and take responsibility for the learning processes and outcomes” (Amerstorfer, 2020, p. 85). They set their own learning goals and adjust the focus according to their needs and interests. For instance, individuals choose which specific topics to research in between class meetings in order to contribute to cooperative problem-solving. Mercer and Dörnyei (2020) concur that choices and the ability to freely express one’s opinion give learners a sense of agency. Furthermore, students enjoy increased autonomy in PBL through the opportunity to adapt the syllabus according to new interests that surface or develop during the course (Amerstorfer, 2020). The high degrees of self-direction and autonomy in PBL create a sense of meaningfulness, which nurtures academic engagement.

Despite increased individual autonomy and self-regulation, the students work together as a team. They set group targets, which can be achieved by synchronizing the efforts of individuals. All students fulfill important tasks in the specific roles they embody, which generates a sense of co-dependency and togetherness (Amerstorfer, 2020). The students identify the exact learning objectives they wish to pursue and follow a clearly prescribed chain of actions to achieve these goals. “[H]aving clear guidelines and transparent learning objectives, accompanied by an outline of possible steps to be taken to complete the task, can notably facilitate the learning process and learner engagement” (Mercer and Dörnyei, 2020, p. 48).

## Research Design

### Research Questions

The research questions (RQs) relate to an undergraduate preservice teacher education course (see section “Research Environment”). Initially, this study was planned to answer two research questions related to aspects of academic engagement (RQ1) and attributes and actions of teachers (RQ2). During data analysis, a third question emerged about how PBL influences engagement and student-teacher relationships, which has not been addressed in the academic literature (RQ3).

RQ1: How do the students perceive their own academic engagement in the PBL course in comparison to other courses?
RQ2: How do the students perceive teacher caring, credibility, feedback, and communication style in the PBL course?
RQ3: How does the PBL approach shape the students’ academic engagement and their relationships with the course instructors?

### Research Environment

The study was conducted in a PBL course in an undergraduate teacher education program for preservice teachers of EFL. The course was comprised of 15 weekly class meetings of 90 min each. The course materials were specifically designed to suit the PBL methodology and reflect the learning objectives as stated in the curriculum. The underlying theme of the course was learner diversity and inclusion. Coupled to the university course, the students completed 30 h in a supervised internship at local secondary schools, where they observed and taught (segments of) EFL lessons.

The course assessment was based on two components: (1) the students’ participation and performance during the PBL lessons and (2) a term paper, in which they reflected on their individual learning growth and the PBL approach. At the end of the semester, the students were invited to give feedback on the course materials, the PBL methodology, the teachers’ performance, and anything else they wished to put forward in relation to the course.

Unlike in previous semesters, the course was taught online due to the Covid-19 pandemic. Although remote teaching influenced teaching and learning in general, extensive previous experience with PBL shows that transferring the course into a virtual classroom did not affect the course materials and had only little impact on the overall PBL set-up. Instead of meeting in an actual classroom, the course was held in a virtual classroom. The roles of individuals in PBL remained the same, as did the 7-step approach to problem solving. Activities in small groups took place in virtual breakout rooms. All students were given moderator rights, which enabled them to use all functions available in the virtual classroom (e.g., screen sharing, breakout rooms, etc.).

### Participants in the Study

The PBL course was attended by 49 English majors in the 5^*th*^ semester of the teacher education program. To adhere to the maximum group size of 15, the students were divided into four parallel groups (A–D), which were taught by three teachers. Out of the 49 students in total, 34 participated in the study (see Table 1).

**TABLE 1:** Overview of PBL groups, teachers, students, and participants in the study.

Group	Teacher	Total number of students	Study participants
A	Lead author	13	11
B	Co-author	14	7
C	Co-author	12	8
D	Anonymous	10	8
***Total***		**49**	**34**

### Data Collection

A short questionnaire was developed to investigate

• the participants’ perceived engagement represented by their perceived effort, dedication, and learning growth in the PBL course compared to other courses they attended in the same semester; and
• the participants’ perception of their teachers’ credibility, caring, feedback, and communication style.

To gain a general overview of the participants’ subjective perceptions, we designed seven statements to be answered with either a *true* or *not true* response. These statements were each followed by an open-ended question or imperative statement to elicit further explanations (e.g., *Please explain why you put/didn’t put more effort into this course.*) and examples of teacher behavior (e.g., *What did/didn’t you appreciate about the teacher’s feedback?*). These follow-up items were aimed at gaining deep insights into the participants’ individual opinions, views, and experiences. The complete questionnaire is included in Appendix 1. Questions 1–3 address aspects of academic engagement (effort, dedication, learning growth). Questions 4–7 address attributes and actions of the teacher (credibility, caring, feedback, communication style).

The participants were informed that their participation in the study was voluntary, that it did not affect their grades for the course, and that the responses would be anonymized and exclusively used for academic research, revision of the course materials, and professional development. The questionnaire was administered online at the end of the final class meeting in all four groups. Students who were absent during the lesson received the link to the questionnaire via email and were invited to respond in their free time.

### Data Analysis

The data analysis was focused on the qualitative information expressed in the participants’ responses to the questionnaire in order to learn about the perceptions of individuals (see section “Qualitative results”). Additionally, percentages of the quantitative data were calculated to provide a general overview of the information (section “Quantitative results”).

The analysis of the qualitative data was a comprehensive process, in which the authors continuously contrasted and complemented each other’s work. First, they discussed how the data can be objectively interpreted and decided to cross-check each other’s work at three stages in the data analysis to increase reliability. After reading through all qualitative data individually, they each determined code definitions and selection criteria, which they then refined together (reliability check 1). Then they independently coded the data and jointly reviewed their findings again (reliability check 2). They independently interpreted the information by comparing individual responses and emerging themes with the academic literature. Finally, they collaboratively scrutinized their individual results (reliability check 3) and formulated the final results. During the interpretation of the data, the authors consulted with the third course instructor on several occasions to include an additional perspective.

## Results

### Quantitative Results

The quantitative results obtained with the *true/not true* statements indicate the participants’ general perceptions of aspects of their academic engagement (effort, dedication, learning growth) and the teachers’ behavior (caring, feedback, communication style, credibility) in the PBL course. Table 2 summarizes the quantitative responses by the participants (p.).

**TABLE 2:**
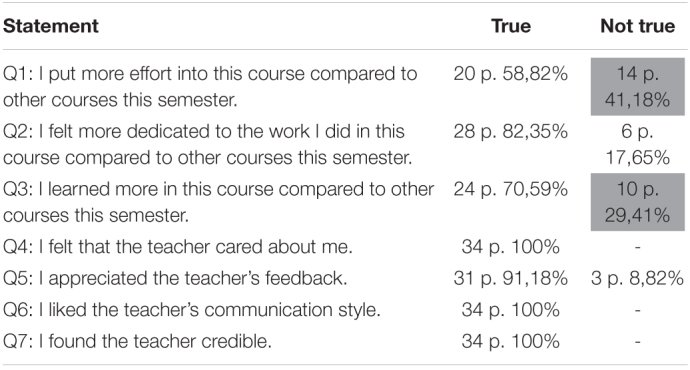
Overview of quantitative results.

*^1^Analyzing student engagement in relation with academic achievement in the transition from high school to post-secondary schooling, Finn and Zimmer (2013) distinguish four components of engagement: academic (students’ observable participation during class), social (how students behave in the classroom), cognitive (brain activity during thought processes), and affective (emotional responses).*

*^2^Participant identification code.*

*^3^Number of participants who provided the response.*

*The column Not true is highlighted in gray for questions 1 and 3 due to the participants’ not wanting to choose either of the two options.*

The open-ended follow-up items revealed that some participants would have preferred a third option in addition to *true* and *not true* because neither answer applied to them. This was the case for four participants in question 1 (the statement was actually *not true* for 10 participants instead of 14) and six participants in question 3 (the statement was actually *not true* for four participants instead of 10). The column *Not true* is highlighted in gray for questions 1 and 3 due to the participants’ not wanting to choose either of the two options.

### Qualitative Results

The following are summaries of the explanations related to the *true/not true* responses gained with open-ended questions and imperative statements.

*1: I put more effort into this course compared to other courses this semester. (true/not true)*

*Please explain why you put/didn’t put more effort into this course.*

An increased effort invested in the PBL course was mainly induced by the course contents and the PBL methodology. In comparison to other university courses, the participants experienced the effort in the PBL course as higher because of the rotating roles and the home assignments, which they set for themselves. Similar to other courses, the term paper at the end further added to the experienced effort. One participant [PB4]^2^ wrote,

A significant amount of effort was put into the home assignments and preparation for each week’s class plus the work put into the assignments when being a scribe or a chairperson. There were some research tasks that also consumed quite a bit of time. Additionally, the effort put into the writing of the final paper cannot be forgotten. Nevertheless, the work resulted in some fruitful insights about language teaching.

Other specific reasons for increased effort in the course were personal interest and enjoyment, relevant topics, the teachers (each mentioned three times), the motivating effect of PBL, and the team spirit created by PBL (each mentioned once). The participants highlighted that the teachers’ encouraging, motivational teaching style, respectful communication, and the effort the teachers put into the course increased their own effort and motivation. Participants responded, for instance, “I put more effort in because I liked the way our professor was teaching and treated us […]” [PD1] and “[…] I appreciate the effort our lecturer was putting into each session what motivated me even more” [PD6].

Four participants indicated that the effort in the PBL course was equal to other courses; yet, one added that PBL “was a little bit easier […] because one learned hand in hand with being scribe and chairperson” and that “remembering information was easier due to that” [PB2]. Two participants equated “effort” with “workload” measurable in ECTS credits. They felt that the actual workload was much higher than the accredited 2 ECTS points, which represent 50 h of study. Three participants explained that their effort was lower than in other courses because “it was a shared experience” [PA11] with shared home assignments and with much learning taking place during group discussions in class. One of them further acknowledged that all effort “was worth it” because it was “helpful for our future life as a teacher” [PA10]. One participant did not explain why they clicked on *not true*.

Some participants’ reflections of their efforts were related to the fact that all university courses were conducted online during that semester. Three participants, for example, used Question 1 as an opportunity to complain about other courses, which they found overwhelming because of too much self-study. This was assumed by the participants to be due to the teachers’ lack of experience with remote teaching. One participant noted that despite the interesting course contents, they would have invested more effort if the course had not been online because they found it difficult to interact with others.

*2: I felt more dedicated to the work I did in this course compared to other courses this semester. (true/not true)*

*Please explain why you felt more/less dedicated to the work in this course.*

Participants who felt more dedicated than in other courses explained that this was because of the interesting topics (3)^3^ that were covered and the PBL design (2). They highlighted that the practical connection to their future careers as language teachers (9) and the internship (6) made the course more relevant for them, which enhanced their dedication. The intense peer-interaction (5) was appreciated, especially due to an overall lack thereof in times of distance learning. One participant [PB7] wrote,

We actually had to interact with the other students because of the way this course was held and this was really good – due to distance learning I kinda lost track of what we are doing in the other classes sind [since] the professors did not really care about interaction in their courses.

Another reason for increased dedication was that the course provided a platform for sharing research findings (4) and that it demanded active participation and interaction (3). The relevance of the individuals’ achievements for the group (4) and not wanting to lose face in front of peers (1) were also incentives for increased dedication. Additionally, one participant highlighted the transparent goals in PBL as encouraging for student dedication. Another participant “felt dedicated to contribute to avoid silence in class” [PC4]. Furthermore, the teachers and the information they provided (2) were named as reasons for increased dedication, as well as experienced pressure to finish the term paper on time (1). One participant was more dedicated because they wanted to profoundly understand the PBL methodology.

Out of the six participants who felt less dedicated in the PBL course, one found some topics redundant and noted that speaking about them in class created more confusion than clarity. Another participant found the repeated procedure during the PBL class meetings boring, which decreased interest and consequently also dedication. One participant did not feel more dedicated in comparison to other courses but emphasized how important participation was during the PBL class meetings. One participant did not explain why they felt less dedicated. Two named the Covid-19 pandemic and the brought about online instruction as the main cause. They found the overall circumstances and remote learning demotivating. One explained, “Corona is siphoning away at all our energies, manny [*sic*.] people (including myslef) [*sic*.] have gotten and still are depressed thus reducing all motivation and incentive to do anything” [PA6].

*3: I learned more in this course compared to other courses this semester. (true/not true)*

*Please explain why you learned more/less in this course.*

Nine participants answered that they learned more in this course because of the practical, interesting topics related to their future profession. The PBL design was highlighted 11 times in regard to learning gain, specifically the close-to-life problem scenarios; the group discussions to share ideas, thoughts, and research findings (each mentioned four times); overall participation and interaction; as well as intense preparation (each mentioned twice). Participants explained, for instance, “I learned more without having studied for it. It was the format of the teaching (problem-based learning) that helped to acquire a lot of information during class through discussions and exchange of information […]” [PA11] and “[…] All the scenarios concerned me personally and my future job and they were interesting. Because these were real scenarios, which partially I have experienced myself already, I learned even more” [PA4]. Other factors that contributed positively to the participants’ perceived learning growth were self-regulated learning processes (2), which the participants experienced as supportive to memorize new knowledge, and the variety of information sources (1).

Two participants who clicked *not true* did not add any comments. Two participants stated that they learned nothing new because they were already familiar with the topics. The remaining six who responded with *not true* explained that the perceived learning gain was similar but the learning experience was not comparable to other courses. One participant [PA8] contrasted PBL with other teaching approaches:

I would not say that I have learned more because in the other courses there was also a lot of imput [*sic*.] but it was presented in different ways for example the professor talked a lot and explained things or there were only power point slides with an audio where the professor talks the students through the slides. I would say I learned in other courses as much as in this course just differently.

Others pointed out that the course contents were different from any other courses and that it was easier to memorize information in the PBL course (each mentioned once).

*Q4: I felt that the teacher cared about me. (true/not true)*

*What makes you think that the teacher cared/didn’t care about you?*

All participants felt that the teacher cared about them. In the explanations, the following adjectives were used to describe caring teachers: understanding (5), respectful, polite, dedicated, friendly, kind (each mentioned twice), fair, empathetic, and well-prepared (each mentioned once). The teachers were further perceived as caring because they provided support (8); showed interest in the students’ lives beyond the classroom; answered all questions; fostered learner engagement and learning gains (each mentioned five times); created a positive learning environment; provided useful advice (each mentioned four times); encouraged student interaction; responded fast to emails; were engaged in the lessons themselves; and provided constructive feedback (each mentioned twice). One participant [PA6] gave a specific example:

When I was not feeling well and did not go to class the teacher reached out to me and asked me whats wrong why I was not participating anymore, i felt really nice then, and thats quintessential for a teacher IMO [in my opinion]. Showing care and understanding for bad things going on is [a] skill we have to possess as teachers. That way we can react accordingly choosing the best approach to make the situation better.

*Q5: I appreciated the teacher’s feedback. (true/not true)*

*What did/didn’t you appreciate about the teacher’s feedback?*

Participants were overall grateful for being provided with feedback at all, understood that the feedback was supposed to help them improve their skills, and appreciated the teachers’ honesty. Many participants found the teachers’ feedback constructive (10), as exemplified in their responses, for instance, “The feedbach [*sic*.] was postive [*sic*.] and constructive, the two elements that make it easy to improve without losing any motivation throughout the semester” [PD3] and “I generally appreciate any feedback, positive or negative. We received a lot of positive feedback from her which was really motivating. Also constructive feedback helped to further myself” [PA4].

Other adjectives attributed to the teachers’ feedback were positive, motivating (each mentioned three times), respectful, extensive, precise, honest, informative (each mentioned twice), polite, objective, product-oriented, immediate, justified, formal, clear, educated, supportive, appreciative, and kind (each mentioned once).

Three participants did not appreciate the teachers’ feedback. One could not remember any specific feedback, and another one did not find the teacher’s feedback constructive. The third one noted just some remarks, which they “liked very much but sometimes critique seemed to be based on personal expectations” [PA5].

*Q6: I liked the teacher’s communication style. (true/not true)*

*What did/didn’t you like about the teacher’s communication style?*

All participants liked the teachers’ communication style, particularly, being treated in a respectful manner. They felt that the teachers had good social skills and experienced them as respectful (4), straightforward, honest, nice, helpful (each mentioned twice), authentic, understanding, and less strict or formal (each mentioned once) compared to university teachers in other courses. The teachers’ communication style was described as friendly (3), clear, polite (each mentioned twice), transparent, open, fresh, motivating, appropriate, formal (each mentioned once), and “sometimes hesitant” [PB1]. Some participants remarked that their teacher had a good sense of humor (2), spoke in a pleasant voice, and was easy to understand (each mentioned once). They appreciated that the teachers focused on the subject matter and created a comfortable learning atmosphere (each mentioned once). Additionally, they noted that the teachers gave the participants opportunities to speak (2), were interested in their students’ opinions, took time to answer questions, responded promptly, and chose their words carefully (each mentioned once). The participants felt included and that the teachers appreciated their answers (each mentioned once). They liked that the teachers acted as tutors, although one participant would have enjoyed more direction by the teacher, specifically, concerning the home assignments. One participant found the teacher “a bit corny” and “trying too hard” [PA6].

*Q7: I found the teacher credible. (true/not true)*

*What makes you think that the teacher was/wasn’t credible?*

All three teachers were found credible by all participants. The participants’ perceptions were mainly based on the teachers’ competence (14), which included factual and practical knowledge as well as enhanced teaching skills and teaching experience at secondary school. One participant [PA5] explained,

I think the teacher made the impression of having the skills and experience to lead this class (or rather in this case to hand over to us students); the teacher also conveyed an image that she had practical experience as school-teacher and was not only focused on academic aspects of teacher education something which I highly appreciated, especially in context of the content which seemed at first very theoretical but proved much more “hands-on” than expected.

Another one [PD4] said that they found the teacher credible “[b]ecause our teacher recently worked in a school which I always appreciate. Often I feel like university teachers have not been in school for a long time and that they only talk about research outcomes which are often not really feasable [*sic*.].”

In connection to the teachers’ credibility, the participants described them as professional (3), well-prepared, helpful (each mentioned twice), reliable, innovative, good at explaining things, authentic, honest, friendly, genuine, supportive, respectful, calm, meticulous, and caring (each mentioned once). Further features and actions that made the teachers appear as credible were their use of reliable sources (3), willingness to share own experiences, being role models (each mentioned twice), admitting knowledge gaps, having transparent aims, treating problems confidentially, giving useful feedback, and responding to emails (each mentioned once). One participant noted that although the course was demanding, the teacher “only asked things of us which she would be willing to do herself” [PA5]. Two participants remarked that their teacher’s research experience speaks for her credibility.

## Discussion

The study was aimed at the qualitative investigation of students’ perceptions regarding aspects of their academic engagement (effort, dedication, learning gain) as well as attributes and actions concerning the teacher (caring, credibility, feedback, communication) in a preservice teacher education course conducted with PBL. The monotony of continuous remote learning due to the Covid-19 pandemic had a negative impact on students’ overall well-being and attitude toward studying (Amerstorfer, 2021). We presume that the distance learning mode that the pandemic enforced upon us also had a negative impact on the participants’ perception of the PBL methodology. Nevertheless, the study confirmed many of the positive effects of PBL as described in the academic literature (see section “Problem-based learning in foreign language teacher education”) and brought to light some of its drawbacks as well. It revealed new details about the influence of PBL on academic student engagement and student-teacher relationships. The gathered findings can be related to similar educational settings and may therefore be valuable for other teachers and researchers.

### Academic Engagement in Problem-Based Learning

There is much teachers can do to increase the academic engagement of students, for instance, applying an appealing teaching methodology; choosing interesting, meaningful contents; offering authentic tasks with an attractive design; increasing student autonomy and self-regulation; fostering a motivating learning environment; and facilitating peer-support and teamwork (Linnenbrink and Pintrich, 2003; Skinner and Pitzer, 2013; Mercer and Dörnyei, 2020; see section “Academic engagement in tertiary education”). All of this can be implemented in PBL.

The study shows that academic engagement is linked to affective factors that concern the students as individuals (e.g., motivation, enjoyment) and members of a team (e.g., team spirit, fear of losing face). The data gathered in this study seems to suggest that the PBL approach increased the effort and dedication of most participants, which was mainly due to the rotating roles in PBL; the intense homework tasks (which the students themselves determined and allocated among group members); and the motivational effects of the course. Some aspects of the course were perceived as particularly motivating, for instance, teamwork, which increased active participation during class and intensified the dedication of individuals, the time pressure individuals experienced regarding the submission deadline of the term paper, and a professional interest in the PBL methodology.

The results of the study indicate that students realize the benefits of their academic engagement in PBL. The participants felt that self-regulated learning and a variety of information sources made it easier for them to memorize new knowledge. The authentic, interesting topics and the problem-solving set-up made them experience their learning gains as sustainable. They particularly appreciated the practical relevance of the course to real life as well as the transparent learning goals (which they often defined themselves) and learning how to achieve them (by following the step-by-step approach). Not only does the study endorse existing theories about self-regulated learning (e.g., that self-regulation fosters motivation and positive learning outcomes; Mercer et al., 2012; Mercer and Dörnyei, 2020), it also expands them from an individual to a group level.

Nevertheless, the PBL course did not increase the engagement of all students because some did not value the topics discussed or found the repeated step-by-step process for problem solving boring. Although this concerned only a small number of participants, we will try to offer a broader choice of topics in the future and consider including alternative problem-solving approaches to increase the attractiveness of the course design.

### Teacher-Related Aspects Concerning Student Engagement in Problem-Based Learning

Students’ academic engagement depends, among others, on the attributes and actions of the teacher. The responses to the individual questions posed in this study’s questionnaire, which were characterized by much overlap and repetition, revealed a strong dynamic between the aspects of academic engagement and teacher caring, credibility, feedback, and communication style. The gathered data lend support to the fact that PBL establishes opportunities for teachers to display care for their students, for instance, by encouraging interaction, fostering engagement and learning gains, providing support on demand, actively engaging in lessons themselves, creating a positive, relaxed learning environment, showing genuine interest in the students’ lives beyond the classroom, and providing meaningful feedback (Savin-Baden and Howell Major, 2004; Hmelo-Silver and Barrows, 2006; Moust et al., 2007; Amerstorfer, 2020; Christopher et al., 2020).

The teacher’s communication style seems to be a major factor for creating a learning environment in which students like to engage in academic activity. The study shows that a teacher’s communication style discloses information about their behavior and personality and that it is an indicator for caring and credibility. The latter does not only depend on academic education and experience. Not knowing the answers to all questions can increase a teacher’s credibility, as one participant noted. Alongside professionality, positive interpersonal skills and other personality traits (e.g., respectfulness, authenticity) have been highlighted as important aspects of a teacher’s credibility.

Regarding feedback, PBL teachers are very mindful about giving feedback (Savin-Baden and Howell Major, 2004; Moust et al., 2007; Yew and Goh, 2016). The constructivist philosophy underlying PBL expects them to hold back and avoid interfering during class as much as possible. However, when the involvement of the teacher is required, they carefully consider the content of their comments as well as the timing and manner in which they are communicated in order to achieve positive effects. As was rightly remarked by one participant, teacher feedback is based on expectations. This can concern content-related learning objectives in a course, students’ performance regarding the PBL methodology (e.g., preparation for class meetings, teamwork), and interpersonal behavior, such as respectful communication and mutual support in a team. The teacher’s expectations can further concern the whole class as well as individual students and can be based on records or observations of previous performance. It is important that the teacher communicates transparent feedback criteria in an unambiguous manner in order to clarify the feedback framework and to strengthen the effectiveness of the feedback. Overall, the study indicates that students understood and appreciated the teachers’ feedback. It further confirms that genuine, constructive feedback affects academic engagement in a positive way (Hattie, 2003; Gettinger and Walter, 2013; Mercer and Dörnyei, 2020).

### Student-Teacher Relationships in Problem-Based Learning

Generally, the findings of the current study in a PBL context are in line with previous studies about student-teacher relationships in other university contexts (Anderson and Carta-Falsa, 2002; Fitzmaurice, 2008; Komarraju et al., 2010). Influences on student-teacher relationships from both the affective and the support dimension (Hagenauer and Volet, 2014) were evident in the students’ qualitative responses. In a nutshell, the teachers were perceived as likeable people who are eager to support preservice EFL teachers in becoming confident and competent professionals.

The study confirms that students experience the overall PBL setup as more relaxed than lecture-type teaching or other teacher-centered approaches commonly applied in higher education. The hence comfortable learning atmosphere in PBL, its student-centeredness, and the corresponding behavior of PBL teachers promote the development of positive relationships between students and teachers. In PBL, teachers are not perceived as majorly superior to students or as the source of all knowledge. Rather, they function as facilitators or guides (Hmelo-Silver, 2004; Savin-Baden and Howell Major, 2004; Moust et al., 2007; Filipenko and Naslund, 2016; Ansarian and Teoh, 2018), which creates a feeling of closeness and supports positive relationship-building among students and teachers. The PBL teachers were consistently perceived as friendly and supportive while maintaining a high degree of professionality. The participants noticed that the teachers tried to create a learning environment that promotes academic engagement and encourages sustainable learning as a group and individually.

The study reveals that the most important characteristic of a PBL teacher and crucial for the manifestation of positive student-teacher relationships in PBL is respect. Respect was emphasized in relation to all four teacher characteristics and particularly connected with autonomous, self-directed problem-solving and syllabus adaptation. Teacher caring and credibility, which are important ingredients of positive student-teacher relationships (see section “Teacher credibility and caring”), were also highlighted by the participants. They were repeatedly related to one another, which affirms the solid connection between them (Noddings, 1992; Noblit, 1993; Wentzel, 1997; Elizabeth et al., 2008; Pishghadam and Karami, 2017; Duffy, 2018; Pishghadam et al., 2018; Mercer and Dörnyei, 2020), as well as in regard to teacher feedback and communication.

The collected data reveal that mindfulness and approachability are further teacher traits that enhance student-teacher relationships. Teachers can appear approachable in a literal sense (e.g., available via phone, responding to emails) as well as figuratively (e.g., sharing personal anecdotes). They can explicitly communicate a willingness to create and cultivate interpersonal relationships with students or imply the same, for instance, by demonstrating care about the students’ learning for real life, which is the ultimate goal of PBL.

Another important finding regarding relationships is that students perceive their PBL teachers as role models, particularly if the teachers come across as authentic, genuinely interested in individuals, welcoming diversity, and open for information sharing and communication. This insight may have been amplified by the fact that the participants were future teachers themselves. Experiencing what “good” teachers do may have increased their wish to become “good” teachers themselves, which does not only depend on the teacher but also on a multitude of contextual aspects inducing the student-centeredness of the PBL methodology. It seems that the wholehearted trust of the teachers in the students’ abilities signaled to the students that they were perceived as competent, efficient problem-solvers and that their efforts were appreciated. This seems to have nurtured the self-confidence and academic engagement of the students, facilitated mutual respect and trust among them (which had positive ramifications on group dynamics and team efficiency), and contributed to positive student-teacher relationships.

### A Critical Look at the Study and Recommendations for Further Research

The instrument was an online form to collect student feedback, which, in our experience, needs to be simple and quick to fill in. Given the high return rate and the valuable insights we gained about the topics under investigation, we believe that the chosen format of closed-ended statements (to set the participants’ minds on the individual topics and to gain a general direction of the students’ perceptions), followed by open ended items (to obtain deep insights about the perceptions of individuals) was appropriate for the purposes of this study. However, critically scrutinizing the research instrument, we realize that the positively phrased statements in the questionnaire (see Appendix 1) may have been somewhat leading. Moreover, some participants would have preferred additional options to *true* and *not true*. In future studies, we would include a quantitative component that allows for more varied responses (e.g., Likert scale, slider bar).

Other weaknesses of the study arise from the fact that the researchers were the participants’ teachers. Even though taking part in the study was voluntary, anonymous, and online, the participants may have (unconsciously) responded more positively in their feedback than they would have done if the data had been collected by an independent researcher. The positive student-teacher relationships in the PBL course and/or students’ previous experiences with the teacher, which potentially concerns 8 students in group A, may therefore have affected the reliability of the findings. Similarly, having known and worked together with the students for a whole semester may have impaired the objectivity of the researchers during data analysis. The outcomes of the study should hence be viewed with caution because the gathered data may have been undermined by phenomena like researcher’s paradox, subject expectancy, or halo effect, which could be prevented by including a third, independent researcher in the data collection and interpretation.

In retrospect, an overall different setup of the study may have led to clearer results regarding RQ3 as it was sometimes difficult to identify the exact influences on student engagement and student-teacher relationships. An experimental study with two parallel groups taught by the same teacher, who applies PBL in only one of the two groups, could support a clearer distinction between PBL-related and other influencing factors. Ideally, the teacher would not have taught the students before and would be unaware of the aims of the study.

Academic engagement and student-teacher relationships in higher education are generally under-researched topics. The same is true for PBL – in itself and particularly in relation to academic engagement and student-teacher relationships. Questions that require further quantitative, qualitative, and mixed-methods research are, for instance,

• How is academic engagement related to strategic learning?
• How does online teaching influence academic engagement/student-teacher relationships in PBL?
• How can academic engagement and positive student-teacher relationships be fostered in remote PBL?

## Conclusion

In PBL, students do not simply co-exist in the classroom but intensely interact and cooperate with each other. Inspired by realistic problem scenarios, they plan and conduct individual and group activities in order to jointly arrive at commonly acceptable solutions. During the problem-solving processes, students develop knowledge and skills beyond the subject matter, which demands a high degree of engagement of individuals. They adopt specific roles within a team and make constructive contributions toward group goals with the ultimate aim of learning for life in a sustainable manner.

PBL may not be ideal for university students who prefer learning by themselves, need greater teacher guidance, or procedural freedom in problem-solving. However, the study confirms that PBL is overall perceived by students as an enjoyable teaching methodology. It provides a relaxed learning environment in which they enjoy engaging as autonomous actors in authentic, meaningful activities that lead to sustainable learning gains. It further provides conditions that foster positive relationships between students and teachers. Respect was the most crucial criteria mentioned in regard to positive student-teacher relationships. It traversed through the students’ perception of teacher caring, credibility, feedback, and communication style like a golden thread.

## Data Availability Statement

The original contributions presented in the study are included in the article/supplementary material, further inquiries can be directed to the corresponding author.

## Ethics Statement

Ethical review and approval was not required for the study on human participants in accordance with the local legislation and institutional requirements. The patients/participants provided their written informed consent to participate in this study.

## Author Contributions

CA designed the study. CA, CF, and a third person collected the data. Both authors analyzed and interpreted the data, contributed to the composition of drafts, repeatedly revised these drafts, and approved the submitted version.

## Conflict of Interest

The authors declare that the research was conducted in the absence of any commercial or financial relationships that could be construed as a potential conflict of interest.

## Publisher’s Note

All claims expressed in this article are solely those of the authors and do not necessarily represent those of their affiliated organizations, or those of the publisher, the editors and the reviewers. Any product that may be evaluated in this article, or claim that may be made by its manufacturer, is not guaranteed or endorsed by the publisher.

## Appendix 1

**Feedback: Communicative Language Teaching in Practice, WS 2020/21**

This questionnaire inquires about your learning experience in the course “Communicative Language Teaching in Practice.” By filling in this questionnaire, you give the teacher feedback on different aspects related to the course and her teaching. Your anonymous responses have no influence on your grade for this course and will be used for academic research, the revision of the course materials, and professional development.
